# Harlem Medical Association

**Published:** 1891-06

**Authors:** A. H. Leary

**Affiliations:** Secretary


### Society Proceedings.

### HARLEM MEDICAL ASSOCIATION.

Reported by A. H. LEARY, M. D., Secretary.

STATED Meeting, held April 1, 1891.

The President, Dr. E. FRIDENBERG, in the chair.

Dr. W. F. MARTIN presented the following

### REPORT OF TWO CASES OF CROUP.

CASE I.—Girl, *æt.* four and a half years; previously in good health, taken suddenly, January 24, 1891, with dyspnea, rapid and labored breathing, slight sore throat. Examination showed moderate congestion and swelling of the tonsils and pharynx, chest filled with sibilant and sonorous rhonchi; prolonged, loud, pitched breathing. No moist sounds. Temperature not taken. The diagnosis of bronchitis with unusual predominance of spasmodic elements was made, and favorable prognosis given. Afternoon of same day, as I could not be reached and child becoming worse, half a dozen other doctors were called in, all of whom pronounced the case extremely critical, most calling it one of membranous croup, or diphtheria. One of these physicians took temperature per rectum at this time, and found it 103½. I reached house in evening, found dyspnea pronounced, both inspiratory and expiratory, stridulous breathing, hoarse laryngeal cough. No rhonchi in lungs; no membrane in pharynx or on tonsils; no enlarged cervical lymphatic glands. Temperature, 102, per mouth. Child had been vomited, with some relief. Steam inhalations given with additional relief, and small continued doses of antimony and ipecac (aa. gr. $\frac{1}{100}$—q. ½ h.) ordered. Child was watched for several hours, and kept in steam atmosphere; benefit quite pronounced, and continued for rest of night. For next three days child continued aphonic with laryngeal cough, having every day from two to three severe attacks of dyspnea, lasting from fifteen minutes to half an hour, otherwise breathing easily. Some of these attacks were relieved by direct inhalation of vapor, others by vomiting and hot external applications, others by nothing additional to that of which was continually kept up, namely, a moist atmosphere, impregnated with eucalyptol and carbolic acid. During this time temperature ranged about 100. On the morning of the 28th, four days after onset, child had a severe attack of dyspnea, which failed to yield to all means previously successful, and which became steadily worse,—so much so, that intubation was strongly urged, and after much hesitation on the parents' part, it was finally done at one P. M., at a time when the dyspnea was extreme. During the morning the temperature, per rectum, was taken and found to be normal. Relief was immediate and lasting. Temperature rose in P. M. to 102½. Next day, temperature normal. Lungs, clear; tonsils and pharynx continued clear; no enlargement of lymphatic glands of neck; cough brought up only mucous expectoration. On the third day following intubation, tube was coughed up. Child made a rapid recovery.

Temperature reached highest point on day of onset of disease, namely, in the P. M. 103½, per rectum. On the fourth day, when intubation was done, it was normal, per rectum, previous to intubation. Pulse varied with temperature and with attacks of dyspnea, from 120 to 160. Respirations were slightly accelerated ordinarily, and ran up to fifty and sixty during attacks of dyspnea. There were distinct attacks of labored breathing, occurring about two to three times in twenty-four hours, and between these attacks quiet respirations were present.

In above case at no time was membrane to be found on tonsils, soft palate, uvula or pharynx. At no time were any enlarged lymphatic glands to be felt in the neck.

Query: Was the case one of diphtheria of the larynx, simple catarrh of the larynx, associated with marked spasm, or something else?

As a supplement to above case, I would mention one in which symptoms were somewhat similar, but of which I have not full details :

Case II.—The case was one in child some two years older than above, in which very pronounced dyspnea recurred nightly for four nights, with quiet and fairly easy breathing during day. No membrane in throat, no glandular enlargement, and no fever, at least none on fourth day. On the fifth night, dyspnea, etc., became so extreme that, failing in the use of antispasmodic and relaxing remedies of all kinds, intubation was performed by Dr. L. Emmett Holt of this city. Immediate relief ensued. Tube was extracted three or four days later, and no further trouble, save that of aphonia, which continued for some weeks afterward.

Case III.—In contrast to above cases was a case of pronounced diphtheria from the start. Child, male, seven and a half years old ; previous good health. When first seen had patch of grayish membrane on right tonsil, enlarged lymphatic glands on right side of neck. Temperature, 100. Had been complaining of slight sore throat for two days previous. Child partially aphonic. Next day, membrane had spread to anterior pillar of fauces and soft palate ; aphonia. Complete dyspnea had set in and steadily increased ; glands increased in size. Temperature had risen to 102. Very transient improvement brought about by emetics. Steam inhalations constantly kept up from the beginning. Dyspnea became excessive, both inspiratory and expiratory. Stridor marked, no vesicular murmur, and intubation finally done, with marked relief. Child coughed up tube three days after, and with it coughed up a cast of upper part of trachea. Child, after this, made an uninterrupted recovery. Main points in this case, *in contrast* with preceding, are : (1.) Membrane on tonsil and spreading, and membrane coughed up. (2.) Constant low temperature, but slowly increasing from 100 to 102, and after intubation reaching 105. (3.) Lymphatic glands of neck enlarged and tender. (4.) Progressive dyspnea, with no marked remissions.

DISCUSSION ON DR. MARTIN'S CASES.

Dr. E. Mayer: I had an attack of acute laryngitis with a dry cough and a feeling of impending suffocation. The larynx being examined showed nothing abnormal beyond extreme redness. Hot steam inhalations and muriate of ammonia effected a cure. I would suggest as a diagnosis in Dr. Martin's first case that it was one of edema of the larynx. Dr. Schreider's tubes are found to be very beneficial in edema of the larynx. They are made of rubber of different sizes and of the length of a urethral catheter. These are allowed to remain in the larynx a varied length of time.

No irritation is produced by this intubation. I use the mirror to introduce the Dwyer or Schreider tube. I had a case of laryngeal stenosis, due to diphtheria, and introduced Dwyer's tube to great advantage. After the fifth day I proceeded to remove it by the extraction, but failed after several attempts. The child was being held by a stranger and was much frightened. It immediately went into convulsions and died. I can give no satisfactory explanation of this result. There has been a good deal said against the Dwyer extractor—some operators refusing to use it, depending on emetics and external manipulation.

Dr. Van Santvoord: It is my opinion that Dr. Martin's first case was one of catarrh of the larynx. This is contrary to my experience, however, in the greater majority of cases. I have examined as many as a dozen cases, post mortem, and found membrane in the larynx, but none was observed in the pharynx during life. I have seen three cases of intubation during measles where it was supposed only catarrh or edema existed, as no membrane could be seen in the pharynx. But autopsy proved membrane to be present in the larynx. These cases are important, because they should be isolated with greater care. After intubation with Dwyer's tube, much benefit is derived by feeding the child with the head held lower than the body.

Dr. Stanton: Does Dr. Mayer use the mirror to aid in removing the tube with the extractor?

Dr. Mayer: Yes.

Dr. Stanton: I do not see how the epiglottis can be managed so as to use the mirror, as this organ is so often swollen and covered with membrane. Every practitioner has his own method in introducing the tube. I always find the epiglottis with the index finger, and use that as a land mark. I have operated on fifty cases. I am not in favor of carrying the tube so far posteriorly in the larynx as advocated by Dr. Martin—it may scrape off the membrane and close over the end of the tube. I have not heard of the objections against the extractor mentioned by Dr. Mayer. Formerly I used the old style extractor which permitted the jaws to be widely dilated; with this a singular accident happened to me. I tried twice to extract a tube, but failed. The character of the breathing immediately changed and gave the impression that the tube had been pushed below the larynx. Dr. Dwyer saw the case and very rapidly and skilfully removed the tube; another was inserted and the child ultimately recovered.

Dr. Geo. H. Cocks: I believe it best always to warn the parents to isolate cases if the symptoms indicate laryngeal stenosis, and these cases should be treated as if they were diphtheria.

Dr. Heineman: The condition of the urine will often aid in the diagnosis between catarrhal and diphtheritic laryngitis.

Dr. E. L. Cocks: I always have the child's arms placed beneath the night dress, which is tightly buttoned, and the limbs of the child held firmly between those of the father holding the child. Thus the child is under perfect control. The head should be thrown far back. The epiglottis should always be felt as a guide. I have had eighteen successful cases in forty-one operations. In some cases I have fed the patient through a soft catheter placed through the nostril. Medicines were thus introduced also. I would like to inquire of Dr. Mayer if he invariably uses the mirror to introduce the O'Dwyer tubes?

Dr. Mayer: No. Only in children who are six or seven years old or older.

Dr. Stanton: The best method of feeding these patients while wearing a laryngeal tube is to lower the head. Even water or medicines should be thus given. I had two patients who complained of pain in the ear after swallowing in this position.

Dr. Martin: I have heard of some objections to the use of the extractor. The tube can be extracted in an emergency by placing the end of the thumb under the lower point of the tube and pressing upward while the tip of the index finger is in the mouth over the upper end of the tube.

### REPORTS OF CASES.

Dr. Franklin: I have a case of severe vomiting of pregnancy. The patient is a primipara, three months pregnant. The emesis has existed some seven weeks; she has been under my care ten days. A few drops of water will often excite vomiting. Even talking will frequently bring on an attack. The uterus is slightly retroflexed and retroverted as well as somewhat prolapsed. I have endeavored to keep the organ in position with cotton, but the vomiting has not been relieved by any kind of medication that I have used.

Dr. Mayer: I would suggest the use of ice to the spine—it has been beneficial in my practice.

Dr. Stanton: In three severe cases, which came under my care and which did not yield to any internal treatment, I stretched the os. One case was relieved, the other two were not.

Dr. E. L. Cocks: I have used cocaine internally as well as chloroform to good effect. That is a curious condition in which the husband vomits during the wife's pregnancy. I was the victim of this condition during the first three months of my wife's first pregnancy. No form of treatment was of value to check the vomiting. Nearly every morning for three months I vomited, but my wife was not sick at all.

Dr. M. C. O'Brien: I have one case to relate which will substantiate Dr. Cocks' condition. My patient was accustomed to indulge freely in alcohol, and I thought his vomiting proceeded from a chronic gastritis. I washed out his stomach frequently for three months, when his vomiting ceased. Internal treatment did no good. One year later the same condition returned with the same history. Twelve months later the vomiting occurred again every morning. I now learned that his wife was pregnant and had been pregnant during the two previous attacks he had had; no treatment was instituted, and he recovered when his wife had finished the three months of gestation. The wife had no nausea or vomiting at all and was perfectly healthy.

Dr. Van Santvoord: One very severe case came under my notice which finally ended in abortion at the sixth month. I did not distend the os, but did try nearly everything known. Rectal injections of bromide and chloral did the most good.

Dr. Martin: I saw a case in the practice of Dr. Wyle that was very severe and had not yielded to any medication. The external os was dilated nearly one and one-half inches, with very satisfactory results, and the woman went on to full term.

Dr. Fridenberg: Some years ago a woman pregnant the fourth time came under my care who vomited excessively. No treatment was of avail. Consultation was held with Dr. McLean, and under his observation and guidance the external os was dilated; unfortunately miscarriage resulted.

---

The Chicago Medical Society has come out with an official organ. We trust that the music furnished by the organ-grinder will receive Keener appreciation than is generally given such efforts.—*Medical Mirror.*

